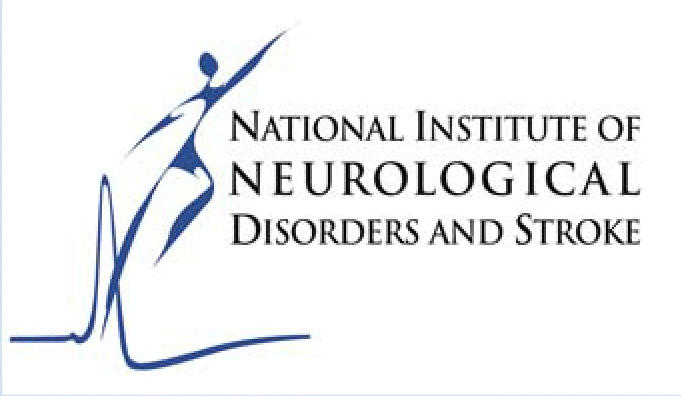# EHPnet: National Institute of Neurological Disorders and Stroke

**Published:** 2006-02

**Authors:** Erin E. Dooley

More than 600 disorders affect the nervous system, and neurological disorders strike an estimated 50 million Americans annually. The National Institute of Neurological Disorders and Stroke (NINDS) is the NIH institute charged with overseeing research on these conditions. The NINDS website at http://www.ninds.nih.gov/ provides the latest news concerning the institute, its programs, and neurological science in general, as well as a resource on the entire spectrum of neurological diseases.

At the top of the homepage is a Disorder Index of the many neurological conditions that the NINDS addresses. Selecting one of the hundreds of disorders takes visitors to in-depth information about the disorder’s symptoms, methods of diagnosis, treatment options, research being done on the disorder, organizations devoted to the disorder, related NINDS publications (including information in Spanish) and additional resources from MedlinePlus.

The site’s homepage features the latest news about neurological diseases, with an archive of older items. The homepage also includes a section listing studies that are seeking subjects. Here, visitors can learn more about what clinical trials really are; those who wish to participate in a clinical trial can choose from an extensive list of neurological conditions, from ADHD to Zellweger syndrome, to see what research is in progress or coming up at the NINDS and elsewhere across the country.

Two sections of the homepage allow researchers to find out about funding opportunities. Under the Funding Opportunities header, visitors can retrieve lists of NINDS opportunities either for the last 60 days or all current opportunities. This section also has information on electronic submission of grant applications and answers questions potential grantees may have about new government requirements for submitting grant applications online. Under the Funding Newsletters header, visitors can sign up for the free *NINDS Notes* newsletter, published three times a year, which contains information on grant applications, requests for applications, and studies that need volunteers. The most current newsletter, plus links to previous editions, are available on the site.

The Neuroscience at NIH section of the homepage contains three sections. The Research at NINDS (Intramural) section contains links to information about NINDS faculty, research facilities, events, and training programs such as summer programs and fellowships. The NIH Blueprint section contains an overview of and link to an NIH framework “to enhance cooperative activities among fifteen NIH Institutes and Centers that support research on the nervous system.” This section also has links to requests for information and requests for applications related to the blueprint. Finally, the Neuroscience@NIH section contains information about NIH neuroscience faculty, areas of research interest, seminars, interest groups, and postdoctoral openings.

## Figures and Tables

**Figure:**